# Understanding the Barriers to Prostate Cancer Population-Based Early Detection Programs: The PRAISE-U BEST Survey

**DOI:** 10.3390/jpm14070751

**Published:** 2024-07-15

**Authors:** Katharina Beyer, Renée C. A. Leenen, Lionne D. F. Venderbos, Jozien Helleman, Sebastiaan Remmers, Vera Vasilyeva, Juan Gomez Rivas, Erik Briers, Thomas Frese, Josep Vilaseca, Shlomo Vinker, Renata Chloupkova, Ondrej Majek, Lieven Annemans, Pieter Vynckier, Partha Basu, Arunah Chandran, Roderick van den Bergh, Sarah Collen, Hendrik van Poppel, Monique J. Roobol, on behalf of the PRAISE-U Consortium

**Affiliations:** 1 Department of Urology, Erasmus MC Cancer Institute, University Medical Center Rotterdam, 3015 Rotterdam, The Netherlands; l.venderbos@erasmusmc.nl (L.D.F.V.); s.remmers@erasmusmc.nl (S.R.);; 2 European Association of Urology, Policy Office, 6842 Arnhem, The Netherlands; 3 Department of Urology, Clínico San Carlos University Hospital, 28040 Madrid, Spain; 4 Europa Uomo, 2018 Antwerp, Belgium; 5 WONCA Europe, Institute for Development of Family Medicine, 1000 Ljubljana, Slovenia; 6 National Screening Centre, Institute of Health Information and Statistics of the Czech Republic, 128 01 Prague, Czech Republic; 7 Institute of Biostatistics and Analyses, Faculty of Medicine, Masaryk University, 625 00 Brno, Czech Republic; 8 Department of Public Health and Primary Care, Interuniversity Centre for Health Economics Research, Ghent University, 9000 Gent, Belgium; 9 International Agency for Research on Cancer, World Health Organization, 90627 69366 Lyon, France; 10 Department of Urology, Gasthuisberg University Hospital, Katholieke Universiteit Leuven, 3000 Leuven, Belgium

**Keywords:** barriers, early detection, population-based screening, prostate cancer

## Abstract

In 2022, the European Commission updated its recommendation on cancer screening, inviting the Member States (MSs) to explore the feasibility of stepwise implementation of population-based screening for prostate cancer (PCa). In line with this recommendation, the PRAISE-U (Prostate Cancer Awareness and Initiative for Screening in the European Union (EU)) project was initiated. As part of the PRAISE-U, we aim to understand the current practice towards early detection in the EU MSs, the barriers to implementing or planning population-based screening programmes, and potential solutions to overcome these barriers. Methods: We adapted the Barriers to Effective Screening Tool (BEST) survey to the PCa context. However, it has not been validated in this context. We translated it into all spoken languages in the EU27 and disseminated it to different stakeholders across the EU using a snowballing approach. Results: We received 410 responses from 55 countries, of which 301 (73%) were from the 27 EU MSs. The most represented stakeholder group was urologists (218 (54%)), followed by general practitioners (GPs) (83 (21%)), patient representatives (35 (9%)), policy stakeholders (27 (7%)), researchers (23 (6%)), oncologists, pathologists, radiologists, nurses, and others (16 (4%)) and one industry representative. Among all respondents, 286 (69%) reported the absence of a population-based screening programme, mainly attributed to resource limitations and a lack of political and medical society support. Out of these 286 respondents, 196 (69%) indicated that opportunistic screening is being applied in their country, and 199 (70%) expressed their support for population-based screening programmes (which was highest amongst patient representatives and urologists and lowest amongst GPs and policy stakeholders). The highest scored barriers were lack of political support, insufficient operational resources, and inadequate participation. Suggested solutions to overcome these included awareness campaigns, consensus meetings, political lobbying and European guidelines (to overcome political support barriers), compatible IT systems (to overcome operational barriers), and easy access (to overcome participation barriers). Conclusions: Participants have noted the presence of opportunistic screening, and particularly urologists and patient representatives expressed their support for the establishment of a population-based PCa screening programme. Nevertheless, successful implementation of population-based screening programmes is complex; it requires political and medical society support, operational resources and capacity, awareness campaigns, as well as the development of protocols, guidelines, and legal frameworks.

## 1. Introduction

Prostate cancer (PCa) screening is one of the most controversial topics in the urological literature [1,2,3]. The introduction of the use of the prostate-specific antigen (PSA) test in 1987 as a screening tumour marker increased the incidence of PCa [4] but also initiated three decades of debate about the specificity of the PSA test and associated overdiagnosis and overtreatment [5,6,7,8].

Concerns about the harm associated with PSA-based screening have hindered the adoption of organised, population-based screening programmes for PCa. Instead, shared decision-making (SDM) for PCa screening decisions is recommended by most health authorities [9,10]. In practice, many men are getting a PSA test regularly [8,11,12]. However, this is not performed in an organised or population-based way but on an individual case finding basis. This is known as opportunistic screening.

To address the harm associated with PCa screening, research has centred on new strategies, described as smart screening or risk-based approaches [5]. This includes incorporating pre-biopsy risk calculation and establishing a risk-adjusted strategy for early PCa detection in addition to a PSA-threshold-only model [13]. The adoption of active surveillance has been emphasised, breaking the link between diagnosis and treatment and creating an environment to reduce unnecessary definitive treatment [14]. The introduction of multiparametric magnetic resonance imaging (mpMRI) has further improved clinical decision-making regarding biopsy indication [15]. Further research into diagnostic biomarkers has also been pursued [16]. Collectively, these developments signal a positive evolution in the ongoing discussion [5,17].

Considering the described evidence, ongoing opportunistic screening practices, and increasing late diagnosis, the European Commission updated its recommendation on cancer screening in 2022, inviting the Member States to explore pilot programmes and further research to assess the feasibility and effectiveness of an organised screening programme [18,19].

In practice, the process of population-based screening is complex. To ensure successful implementation of a screening programme, it is crucial to map potential barriers at the levels of service users, service providers, and health systems, along with their corresponding needs. As part of the Prostate Cancer Awareness and Initiative for Screening in the European Union (PRAISE-U) project (www.uroweb.org/praise-u, (accessed on 21 May 2024)) [20], we aim to understand the current practice towards early detection and identify and prioritise barriers to population-based screening programmes for PCa, as well as potential solutions to overcome them.

## 2. Materials and Methods

To understand the barriers healthcare systems are facing when implementing or planning to implement population-based early detection programmes, we used the Barriers to Effective Screening Tool (BEST) [21,22], which has been developed to evaluate the components of and barriers to an effective screening system for breast, cervical, and colorectal cancer. We amended the questionnaire to fit the PCa context, since currently, in most countries, an organised population-based screening programme, as it exists, for example, for breast cancer, is not established. The BEST instrument was transferred into an online survey and translated into the languages spoken in the 27 EU MSs (see Supplementary Table S1). The survey was approved by the ethics board of Erasmus MC (Ref: MEC-2023-0358).

### 2.1. The Barriers to Effective Screening Tool (BEST)

The EU-TOPIA (TOwards imProved screening for breast, cervical, and colorectal cancer In All of Europe) project developed the BEST instrument following a complex healthcare system approach [22]. The instrument includes two sections to evaluate barriers to effective screening. They used conceptual models to develop the two sections and divided them into six subsections: knowledge generation, identification of the eligible population, maximising uptake (informed participation), successful operation of the programme, adequate follow-up, and effective treatment for those who need it. The six subcategories include different barriers which were validated in a pilot project and an extension of the pilot project [22].

Due to the different context of PCa early detection, which is less advanced in implementation, we amended the BEST instrument to fit into the PCa context. Table S1 in the Supplementary Materials highlights the changes applied.

Following the amendments to fit the PCa context, twenty-nine different barriers across nine different components could be ranked from zero (“not at all important”) to five (“very important”) and the sum of the scores indicated their priority ranking. Solutions to each barrier could be proposed in open-answer options.

### 2.2. Participant Criteria

Clinicians (urologists and primary care physicians), HCPs (radiologists, pathologists, oncologists, and nurses), representatives of patient organisations, researchers, and policymakers involved in PCa were invited to fill out the survey.

### 2.3. Recruitment and Data Collection

Our recruitment and data collection strategy were threefold: Firstly, we presented the project at the European Association of Urology’s (EAU’s) National Society Meeting in Noordwijk on the 10 June 2023. The first introduction was via a presentation at the General Assembly, followed by four discussion group workshops. Here, participants were asked to act as a representative of their country to use a snowballing approach by inviting their contacts to fill in the survey. Participants of the discussion group meetings received a flyer with a QR code to scan that was linked to the survey. Secondly, we shared a newsletter via the EAU’s PCa network, which introduced the survey and asked participants to take the survey and share it with colleagues. Thirdly, the newsletter was shared with policy networks via the International Agency for Research on Cancer (IARC), with patient representatives via Europa Uomo, and with primary care physicians via the World Organization of Family Doctors. The survey was open for responses from the 10 June until the 26 October 2023.

### 2.4. Data Analysis

Descriptive statistics were performed using R version 4.3.3.

## 3. Results

### 3.1. Sample Characteristics

**Figure 1:**
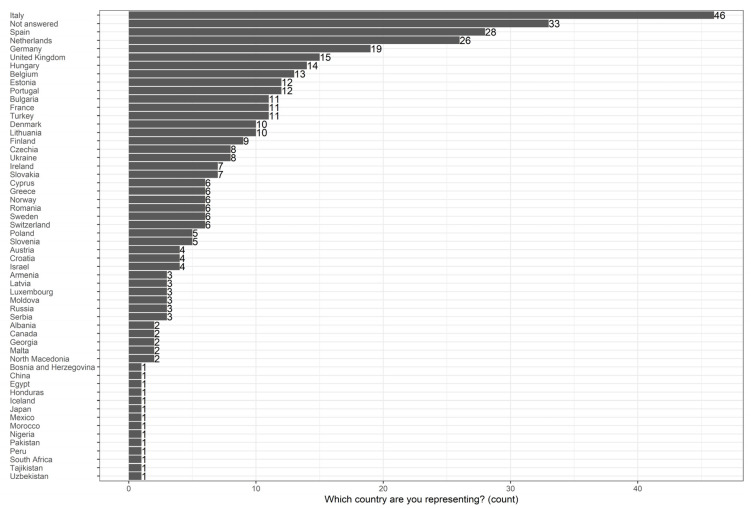
Representative countries.

We received a total of 410 responses from 55 countries, of which 301 (73%) are from respondents indicating an EU Member State (MS). Seventy-six (18%) are from a non-EU MSs, and 33 (8%) did not answer the question and answered in Spanish, Dutch, or English and therefore cannot be assigned to a country as there are several countries where these languages are spoken (see Figure 1).

We received 218 responses from urologists; 83 from primary care physicians; 35 from patients representing a patient organisation; 27 from policy stakeholders; 23 from researchers; 16 from healthcare professionals (HCPs), including nurses, oncologists, pathologists, radiologists, and radiotherapists; and 1 from an industry representative. Seven did not answer the question regarding their occupation.

### 3.2. Presence of Early Detection

#### 3.2.1. Population-Based Screening Programmes

In the first part of the survey, we wanted to understand if there are countries where there is currently a population-based PCa screening programme in operation or one planned for the future (i.e., between 10 June 2023 and June 2024). Out of the 410 respondents, 286 (69%) answered ‘no’, 89 (22%) answered ‘yes’, and 28 (7%) were not aware if their country has a population-based PCa screening programme in place or planned for the future (7 (2%) did not answer the question).

The reasons given for not implementing population-based screening are multifaceted. Examples are linked to a lack of resources, screening tools, screening protocol, evidence base of more benefit than harm, and awareness. In addition, not being a current political priority and therefore receiving limited support and lack of national and medical society consensus resulted in a lack of trust in early detection. Other participants highlighted that the current system is working (i.e., opportunistic screening) or that there is no need due to low incidence and prevalence (see Table 1 for supporting quotes). In Ukraine, the war is impacting progress.

Respondents who had previously answered ‘no’ that there is no population-based PCa screening programme implemented or planned (i.e., 286 participants answered ‘no’) were asked additional questions to understand whether there is a regional, local, or hospital-based PCa screening programme. They were also asked if they believe opportunistic screening is occurring in their country and if they would support the implementation of a population-based PCa screening programme in their country.

#### 3.2.2. Regional, Local, or Hospital-Based Screening Programmes

Out of the 286 respondents (representing those who answered ‘no population-based PCa screening programme is in place/planned’), 237 (83%) answered that there is currently no regional, local, or hospital-based PCa screening programme in place, while 29 (10%) reported a screening programme; however, most screening programmes which respondents reported on did not include PCa (e.g., breast cancer, colon cancer) or are opportunistic, except a trial site or the Swedish population-based programme for organised prostate cancer testing (OPT). Twelve (4%) respondents answered with ‘unknown’, and eight (3%) did not respond to the question.

#### 3.2.3. Opportunistic Screening

Opportunistic screening was reported to be prevalent, as 196 (69%) of the respondents stated that opportunistic screening exists in their country.

To understand which profession reports opportunistic screening, we stratified the number of responses and calculated what percentage of their group they present. Of those who answered the question, 105 (68%) urologists, 33 (62%) primary care physicians, 17 (89%) policy stakeholders, 18 (90%) researchers, 13 (52%) patient representatives, and eight (62%) HCPs reported opportunistic screening (two did not answer the question).

Countries where respondents highlighted the presence of opportunistic screening include Austria, Belgium, Bosnia and Herzegovina, Bulgaria, Canada, China, Croatia, Cyprus, the Czech Republic, Denmark, Estonia, Finland, France, Germany, Hungary, Iceland, Ireland, Israel, Italy, Luxembourg, Malta, Mexico, Moldova, the Netherlands, Norway, Poland, Portugal, Romania, Serbia, Slovakia, Slovenia, South Africa, Spain, Sweden, Switzerland, Turkey, and the United Kingdom.

The opportunistic nature of the screening is attributed to various reasons, such as the clinician’s request for a PSA test as part of a case finding, a man requesting a PSA test, or a shared decision-making environment advocating PSA testing. Moreover, additional factors contributing to the opportunistic approach include the incorporation of PSA testing in occupational assessments, private insurance coverage supporting PSA tests, and inclusion in yearly national health checks.

#### 3.2.4. Support for/against Screening

**Figure 2:**
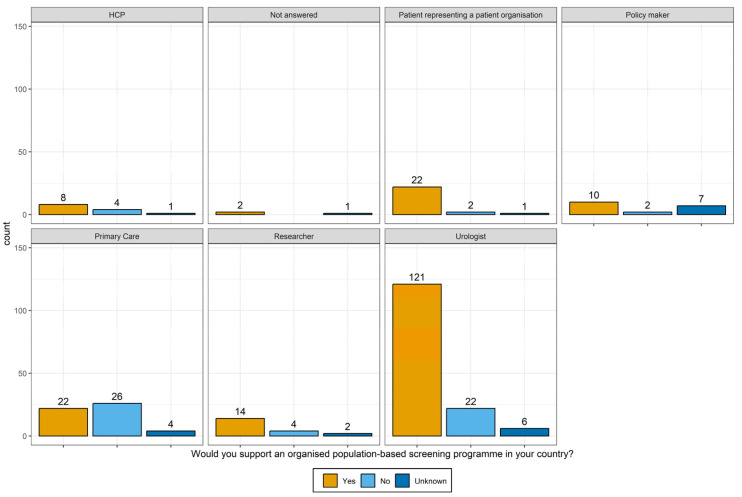
Support for organised population-based screening per job role.

Of the respondents, 70% (199) answered that they would support a population-based PCa screening programme in their country, and 21% (60) would not support it. Figure 2 shows the respondents who support (or do not support) a PCa screening programme, stratified by profession. The question revealed that 22 (88%) out of the patient representatives, 121 (79%) urologists, 14 (70%) researchers, and 8 (62%) HCPs participating in this particular question expressed support for a population-based PCa screening programme.

Reasons for expressing support for a population-based PCa screening programme are the reduction in PCa mortality, an increase in early diagnosis and improved quality of life linked to early diagnosis, patient preference, and reduction in opportunistic screening.

In contrast, only 10 (52%) policymakers and only 22 (42%) primary care physicians expressed their support. The decision not to endorse a population-based PCa screening programme was attributed to various factors, including cost-effectiveness and potential harm outweighing the benefits, highlighting a PSA-to-biopsy algorithm. Additionally, respondents highlighted the healthcare system’s limited capacity and the necessity to allocate resources elsewhere as key considerations in this determination.

The new recommendation by the European Council to further investigate PCa early detection influenced 135 (47%) of the respondents in their opinion to support a population-based PCa screening programme.

### 3.3. Reported Barriers

**Figure 3:**
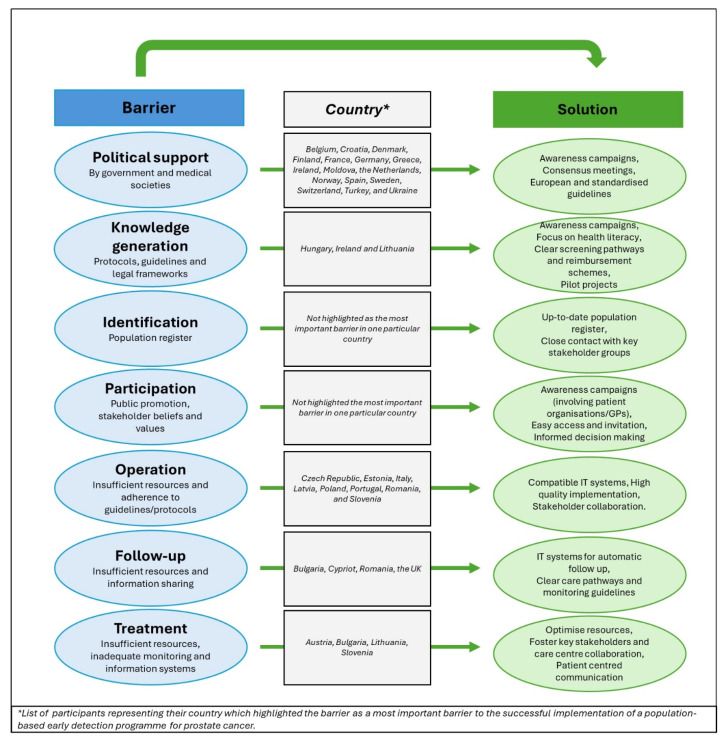
Overview of the identified barriers and proposed solutions to overcome these.

The second part of the survey focused on identifying specific barriers to screening for each of the components previously defined by the EU-TOPIA project and amended by the PRAISE-U. These components include (a) political support; (b) knowledge generation; (c) identification of the eligible population; (d) maximising uptake (informed participation); (e) successful operation of the programme; (f) adequate follow-up; and (g) effective treatment for those in need. An overview of the identified barriers, proposed solutions, and countries were this barrier was ranked most important is provided in Figure 3.

#### 3.3.1. Political Support

Political support, specifically inadequate government support, was identified as the most significant barrier when considering the total ratings from the respondents. Within specific subgroups, urologists, policymakers, and HCPs also emphasised this barrier as the most crucial. In Belgium, Croatia, Denmark, Finland, France, Germany, Greece, Ireland, Moldova, the Netherlands, Spain, Turkey, and Ukraine, this barrier was rated by the respondents as the most crucial (we only included countries with at least four participants’ ratings for the highest-rated item). Patients and primary care physicians rated inadequate support from their respective medical societies for a population-based PCa screening programme as the most important barrier, as did respondents from Greece, Sweden, and Switzerland. Participants from Norway rated both barriers as equally important.

To overcome the political support barrier, respondents shared experiences that were successful in addressing similar challenges in other cancer contexts. They recommended awareness campaigns about the current evidence and how it differs from the existing knowledge in the population, consensus meetings with key stakeholders, political lobbying, close collaboration with medical societies, and the development of European guidelines for screening.

#### 3.3.2. Knowledge

Knowledge, specifically the barrier issues with establishing protocols, processes, and legal frameworks, was highlighted overall as the sixth most important barrier and by urologists as their fifth most important barrier to the implementation of a population-based PCa screening programme and the fourth most important for primary care physicians. Hungary, Ireland, and Lithuania rated this barrier as the most important in their respective countries.

That (screening) guidelines and protocols are not regularly updated or updates are delayed was not rated as a high concern.

To overcome the knowledge barriers, highly successful strategies included a focus on health literacy, especially in minority populations; more extensive awareness campaigns (including on national television); heightened awareness efforts targeting doctors, sharing clear pathways that also highlight the side effects of screening; improving discussions with regulatory authorities; and establishing clear reimbursement schemes and well-established pilot projects in specific countries.

#### 3.3.3. Identification

Identification issues, particularly that the population register is not accurate or complete, are rated as the least important two barriers. Suggestions to overcome these barriers were keeping a population register up to date and ensuring close contact with primary care physicians and urologists.

#### 3.3.4. Participation

Participation, exemplified by inadequate public promotion of the screening programme, was ranked as the third most important barrier by the overall respondents. Specifically, urologists (third), patients (third), policy makers (fourth), primary care physicians (third), and researchers (fourth) considered this barrier significant. Russian and Slovakian respondents highlighted this barrier as the most important.

Patients rated inadequate public promotion as the fourth most important barrier. Other participation barriers were rated to be of lower concern.

To boost participation, respondents primarily recommended awareness campaigns that could reach the target population through media channels, social media platforms, community-driven education, involving patient organisations, and engaging primary care physicians. Respondents also highlighted the importance of overcoming structural barriers and ensuring easy access. For instance, the screening team could visit marginalised communities directly with a ‘screening’ bus or home testing could be provided. Additionally, the target population could be invited through phone calls, messaging apps, or emails. Overall, there was a focus on clear communication and ensuring that men are adequately informed to make an informed choice.

#### 3.3.5. Operation

Operation, particularly the “insufficient human, physical, and/or financial resources to operate a screening programme”, was identified as the second most significant barrier by all respondents. When stratified by subgroups, researchers considered this barrier the most significant, while urologists and policymakers also ranked it as the second most significant obstacle. HCPs identified it as the third most significant barrier. Respondents from the Czech Republic, Estonia, Italy, Poland, Portugal, Romania, and Slovenia highlighted it as the most important barrier. Latvian respondents also identified “Inadequate information technology (IT) systems” as the most significant barrier.

“Inadequate adherence by providers to screening guidelines and protocols” was identified as the fifth biggest barrier by researchers. The other operational barriers seemed less of a concern and were not highlighted in the top five.

Ways to overcome the barrier of operation include implementing a compatible IT system that consolidates information in a national electronic health record. Ensuring high-quality implementation for sustainability, clear documentation, and audit processes is crucial. Additionally, promoting open discussions about the benefits of a risk-based screening approach, strict adherence to screening recommendations, fostering collaboration between stakeholders, and learning from the experience of breast cancer screening are essential measures.

#### 3.3.6. Follow-up

Similar to concerns about the availability of human, physical, and/or financial resources to operate a screening programme, respondents expressed worry about the resources required to conduct follow-up investigations. This concern was rated as the fifth biggest barrier for the overall group, second for primary care physicians and HCPs, fourth for urologists, and fifth for policymakers. It was identified as the most important barrier for respondents from Bulgaria and the UK.

For Cypriot and Romanian respondents, the “Inadequate sharing of follow-up information between national/regional screening organisations, providers of follow-up investigations, and primary care” is a significant concern.

To ensure adequate follow-up, respondents suggested utilising IT systems to support it, such as implementing automatic follow-up algorithms in electronic health records or a central database. Defining clear monitoring guidelines and care pathways and fostering collaboration among key stakeholders are essential as the burden cannot rest solely on one particular group. Adequate funding should also be ensured to support these initiatives.

#### 3.3.7. Treatment

Only HCPs expressed concern about providing treatment, specifically the barrier “Insufficient human, physical, and/or financial resources to provide treatment to those that need it”. They rated this barrier as the fifth biggest obstacle. Slovenia considered it as the most significant barrier.

An “Inadequate system for monitoring treatment information” was identified as the most important barrier in Bulgaria and Austria, and “inadequate sharing of treatment information between national or regional screening organisations, providers of cancer treatment, and primary care” was identified as the most important barrier in Lithuania.

To ensure effective treatment for those who need it, respondents expressed the need to optimise resources, foster collaboration across specialties within care centres, and promote collaboration between care centres (centralised care). Clear, patient-focused communication is crucial, and seeking advice from experiences with breast cancer care was also recommended.

#### 3.3.8. Other

Additional barriers highlighted by the respondents’ included differences in screening criteria between various communities, the absence of an audit system, limited utilisation of multidisciplinary teams, restricted use of active surveillance, and non-adherence to guidelines.

## 4. Discussion

Among the 410 survey respondents from 55 countries, 22% reported existing or planned population-based screening programmes for PCa, while 69% indicated their absence, attributing this to a range of challenges, including resource limitations and a lack of political and medical society support. Opportunistic screening was reported to be prevalent, confirmed by 69% of the 286 respondents (who did not state having a population-based PCa screening programme already in place or planned), with notable variations in reported practices across different countries. The clear majority of patient representatives and urologists support the implementation of a population-based screening programme; however, less than half of primary care physicians are in favour. The three highest-ranked barriers are discussed below.

Political support has emerged as a significant barrier, resonating across diverse subgroups and countries. Survey participants suggest that strategic interventions, such as awareness campaigns, consensus meetings with key stakeholders, political lobbying, close collaboration with medical societies, and the development of European guidelines for screening, are necessary to overcome this obstacle. Currently, countries across the EU are positioning themselves regarding PCa screening, with most either recommending further research or already preparing for the implementation of population-based pilot programmes utilising a risk-based approach [23]. Data from these pilots can serve as the initial step towards understanding the applicability of screening programmes in the respective countries and may facilitate overcoming the barrier. However, there is still an ongoing discussion concerning harm and benefits, which is currently addressed by advocating for risk-adapted screening. Across the EU, different trials are testing different algorithms and are presenting promising results, which steer away from the traditional PSA screening (PSA-to-biopsy algorithm) to a new era of risk-adapted screening approaches, including risk stratification and MRI [24,25,26,27,28].

Operation, particularly the “insufficient human, physical, and/or financial resources to operate a screening programme”, was recognised as the second most critical barrier towards the implementation of a screening programme. This barrier is multifactorial, and participants recommend implementing a compatible IT system, high-quality implementation for sustainability, clear documentation, and audit processes, as well as promoting open discussions about the benefits of a risk-based screening approach and strict adherence to screening recommendations. This concern is shared in the urological literature, especially with a focus on MRI availability. The use of MRI, and particularly multiparametric MRI (mpMRI), is recommended and is an important step in the risk stratification of the newly proposed algorithms. However, access to MRI differs between countries [29]. A new study, ‘the PRIME study’, is trying to tackle this problem by comparing biparametric MRI (bpMRI) to mpMRI, which will ultimately help to understand how to reduce costs and increase accessibility [30].

Participation, exemplified by inadequate public promotion of the screening programme, was ranked as the third most important barrier by the overall respondents. This barrier is twofold: The participation of men in a screening programme, as well as the support of health care providers, e.g., primary physicians. Participation in screening programmes has been a recurring barrier for the last 20 years. When publishing their first results, the European Randomized Study of Screening for Prostate Cancer (ERSPC) study reported that 82% of men accepted at least one offer of screening [31] and the Prostate, Lung, Colorectal, and Ovarian (PLCO) Cancer Screening Trial on PCa mortality reported a compliance rate of 85% [32]. In contrast, the current big screening trials have a much lower participation rate. The ProScreen study has a participation rate of 52% [26,33], the Götenburg-2 trial 47% [24], and PROBASE only 11% [27]. These results are similar to the results of the Swedish regional population-based organized prostate cancer testing (OPT) programmes, which published a participation rate of 35% [34]. These numbers are concerning for ensuring the implementation of a successful screening programme on a population-based level. These low numbers might be linked to the contradictory recommendations of government bodies and medical societies, where a PSA test is not recommended; however, men can request it themselves.

The support of primary care physicians in population-based screening was reported to be low in our study. With little support from this key stakeholder group, it will be difficult to ensure successful implementation. Hence, awareness campaigns and clear communication supported by new data from the ongoing trials and PRAISE-U pilot projects highlight the new risk-adapted approach to screening is important.

### Limitations

A limitation of this study is that the BEST survey has not been validated in the prostate cancer cohort. The BEST tool has been designed and validated in cohorts where population-based screening has been well established in the EU to improve the effectiveness of the screening programme. However, this does not apply to a PCa screening programme, as until now, mostly research studies and pilot programmes have been conducted.

## 5. Conclusions

Participants have noted the presence of opportunistic screening. A majority of the respondents, and particularly urologists and patient representatives, expressed their support for the establishment of a population-based PCa screening programme. Nevertheless, the successful implementation of such a programme requires awareness of risk-adapted screening, political support, support by all key stakeholders involved in screening, and the development of protocols, processes, and legal frameworks with a focus on clear communication. Thirty years of discussion about the harm and benefits of prostate cancer screening created a lot of resistance against PSA screening but also provided a lot of evidence to move forward to a risk-adapted and potential population-based approach.

## Figures and Tables

**Table 1:** Quotes from participants.

“Why does your country not have a population-based screening programme for prostate cancer in operation?”
*“Unfortunately, it is not in focus, lack of money, GPs cannot screen with PSA only urologists”—**Primary care physician, Hungary***
*“Money, bureaucracy, too many other problems in the national health system”—**Urologist, Portugal***
*“Such a programme is not supported by the government, the Health authorities, nor the HCP’s”—**Urologist Norway***
*“lack of capacity to implement multiple population-based screening programs”—**Policy maker, unknown country***
*“Lack of scientific evidence for doing more benefit than harm”—**Urologist Denmark***

## Data Availability

The data is unavailable due to privacy or ethical restrictions.

## Supplementary Materials

The following supporting information can be downloaded at: https://www.mdpi.com/article/10.3390/jpm14070751/s1, Table S1: Amended BEST Survey.
