# Supplementary material for: Understanding the Barriers to Prostate Cancer Population-Based Early Detection Programs: The PRAISE-U BEST Survey
Source: J Pers Med. 2024 Jul 15;14(7):751. doi: 10.3390/jpm14070751 (PMC11277738; doi:10.3390/jpm14070751)
Supplement: Supplementary file 1 [file jpm-14-00751-s001.zip › jpm-3057679-supplementary.pdf]

Supplementary material - Table S1: Amended BEST Survey

Green represents the added questions

Original BEST survey can be found at: https://eu-topia.org/eu-topia-road-map-development/ (accessed at

Question
**1. Which country/region/municipality are you representing? [amend/delete as appropriate for your project]** [ENTER TEXT IN COLUMN B, C ETC]
1.a. If you selected Other, please specify:
**2. What is your job role? [amend/delete as appropriate for your project depending on which stakeholders you are including]** [ENTER TEXT IN COLUMN B, C ETC]
2.a. If you selected Other, please specify: Urologist in Academic Hospital, Urologist in non-academic hospital, Urologist outside of hospital (private practice), primary care, patient representing a patient organisation, representatives from national screening organisations, regional screening organisations cancer registry, epidemiologist, coordinator in the national public health institute, program coordinator, representative from the Ministry of Health, quality assurance and commissioner, other
**3. Is there an organised, population-based screening program for prostate cancer currently in operation or planned for the future (i.e. between now and June 2024) in your country? [If the response is no, please answer 3.1- 3.5, if your answer is yes, go straight to 5] [select yes/no/unknown from dropdown list in column B, C etc]**
**3.1. Why does your country not have a population-based screening programme for prostate cancer in operation?**
**3.2. Does your country have any organised prostate cancer screening programs, such as regional, local, or hospital-based programs? If yes, could you please provide details on the program, including its name, description, and any available references?**
**3.3. Does your country apply opportunitistic screening for prostate cancer?**
**3.4. Would you support an organised population-based screening programme in your country? Please explain why you either support or do not support this.**
**3.5. Does the European Commission recommendation influence your opinion towards organised population based screening programmes?**
**4. How important is each of these barriers to the overall success of an already established or future prostate cancer screening program in your country?**<br>**Rate from 1 (not at all important) to 5 (very important). Score 0 where the barrier does not exist in your country.**<br>**Please conduct this rating exercise for your country alone. Please do not rate the barriers relative to your knowledge of the barriers in other countries. [select rating from dropdown list in column B, C etc] - adapt to potential**
4.0. Political support- No governmental support (e.g. health ministry) for a population based screening programme
4.1. Political support- No support from the respective medical societies for a population based screening programme
4.2. Knowledge - Issues with establishing protocols, processes and legal frameworks (e.g. inadequate national governance structure, professionals with relevant knowledge)

4.3. Knowledge - (Screening*) guidelines and protocols are not regularly updated or updates are delayed (e.g. by complex administration procedures)<br>*if seperate screening guideline exists
4.4. Identification - Population register is not accurate (e.g. not updated with changes of address)
4.5. Identification - Population register is not complete (e.g. some eligible people not included)
4.6. Participation - Some people (e.g. Participant, primary care physicians, Urologist) have beliefs and values that lead to non-participation in screening programme
4.7. Participation - Some people (e.g. Participant, primary care physicians, Urologist) experience practical issues that lead to non-participation in screening programme (e.g. inconvenient appointments, inadequate health insurance)
4.8. Participation - Inadequate public promotion of screening programme (e.g. primary care physicians are not sharing information or promoting screening, shared information does not reach participants adequately)
4.9. Participation - Inadequate reimbursement of screening programme
4.10. Participation - Inadequate system for monitoring levels and patterns of screening participation (e.g. inequalities among some subgroups)
4.11. Participation - (potential) inadequate response to low levels of uptake (informed participation) and patterns of screening participation (e.g. inequalities among some subgroups)
4.12. Operation - Inadequate information technology (IT) systems (e.g. disjointed systems)
4.13. Operation - Insufficient human, physical and/or financial resources to operate screening programme (e.g. limited capacity, organisational or logistical issues)
4.14. Operation - (potential) inadequate adherence by providers to screening guidelines and protocols (e.g. opportunistic screening occurs outside the organised screening programme)
4.15. Operation - (potenital) inadequate collaboration between national or regional screening organisations, providers of cancer treatment and primary care
4.16. Operation - Inadequate system for monitoring operational aspects of the screening programme (e.g. quality of screening experiences of those who participate)
4.16. Operation - Inadequate response to address quality issues relating to the operation of the screening programme
4.17. Follow up - Insufficient human, physical and/or financial resources to conduct follow-up investigations for those that need it
4.18. Follow up - (potential) inadequate adherence by providers to follow-up guidelines and protocols (e.g. clinician's attitudes and established pattern of practice)
4.19. Follow up - (potential) inadequate system for monitoring people who require follow-up investigations but do not participate (e.g. due to personal beliefs or practical issues)
4.20. Follow up - (potential) Inadequate response to people who require follow-up investigations but do not participate (e.g. due to personal beliefs or practical issues)

4.21. Follow up - Inadequate sharing of follow-up information between national/regional screening organisations, providers of follow-up investigations and primary care
4.22. Treatment - Some people have beliefs and values that lead to them declining cancer treatment
4.23. Treatment - Insufficient human, physical and/or financial resources to provide treatment to those that need it
4.24. Treatment - Inadequate system for monitoring treatment information (e.g. treatment data not systematically linked to cancer screening data)
4.25. Treatment - Inadequate sharing of treatment information between national or regional screening organisations, providers of cancer treatment and primary care
4.26. Other- If you experience or know of any other barriers, please specify in the field below.<br>Please include in the ranking.
**7. If appropriate, please provide any further details on the how the barriers (potentially) apply specifically to screening in your country.**[ENTER TEXT IN COLUMN B, C ETC]
**8. Please provide examples of how barriers to population-based screening programs for prostate cancer or other cancer types such as breast and cervical cancer, have been overcome or reduced in your country?**<br>**Please indicate the level of success (low, medium, high) associated with the intervention.**
8.1.a. Political support - Examples of ways to overcome (or reduce) barriers [ENTER TEXT IN COLUMN B, C ETC]
8.1.a. Political support - Examples of ways to overcome (or reduce) barriers [ENTER TEXT IN COLUMN B, C ETC]
8.2.a. Knowledge generation - Examples of ways to overcome (or reduce) barriers [ENTER TEXT IN COLUMN B, C ETC]
8.2.b. Knowledge generation - Level of success of ways to overcome (or reduce) barriers
8.3.a. Identifying the eligible population - Examples of ways to overcome (or reduce) barriers [ENTER TEXT IN COLUMN B, C ETC]
8.3.b. Identifying the eligible population - Level of success of ways to overcome (or reduce) barriers
8.4.a. Maximising uptake (informed participation) - Examples of ways to overcome (or reduce) barriers [ENTER TEXT IN COLUMN B, C ETC]
8.4.b. Maximising uptake (informed participation) - Level of success of ways to overcome (or reduce) barriers
8.5.a. Successful operation of the programme - Examples of ways to overcome (or reduce) barriers [ENTER TEXT IN COLUMN B, C ETC]
8.5.b. Successful operation of the programme - Level of success of ways to overcome (or reduce) barriers
8.6.a. Adequate follow-up - Examples of ways to overcome (or reduce) barriers [ENTER TEXT IN COLUMN B,C ETC]
8.6.b. Adequate follow-up - Level of success of ways to overcome (or reduce) barriers
8.7.a. Effective treatment for those that need it - Examples of ways to overcome (or reduce) barriers [ENTER TEXT IN COLUMN B, C ETC]
8.7.b. Effective treatment for those that need it - Level of success of ways to overcome (or reduce) barriers
